# Physical Analyses of *E. coli* Heteroduplex Recombination Products *In Vivo*: On the Prevalence of 5′ and 3′ Patches

**DOI:** 10.1371/journal.pone.0001242

**Published:** 2007-11-28

**Authors:** Laura M. Gumbiner-Russo, Susan M. Rosenberg

**Affiliations:** 1 Department of Molecular and Human Genetics, Baylor College of Medicine, Houston, Texas, United States of America; 2 Department of Biochemistry and Molecular Biology, Baylor College of Medicine, Houston, Texas, United States of America; 3 Department of Molecular Virology and Microbiology, Baylor College of Medicine, Houston, Texas, United States of America; 4 Dan L. Duncan Cancer Center, Baylor College of Medicine, Houston, Texas, United States of America; University of Massachusetts, United States of America

## Abstract

**Background:**

Homologous recombination in *Escherichia coli* creates patches (non-crossovers) or splices (half crossovers), each of which may have associated heteroduplex DNA. Heteroduplex patches have recombinant DNA in one strand of the duplex, with parental flanking markers. Which DNA strand is exchanged in heteroduplex patches reflects the molecular mechanism of recombination. Several models for the mechanism of *E*. *coli* RecBCD-mediated recombinational double-strand-end (DSE) repair specify that only the 3′-ending strand invades the homologous DNA, forming heteroduplex in that strand. There is, however, *in vivo* evidence that patches are found in both strands.

**Methodology/Principle Findings:**

This paper re-examines heteroduplex-patch-strand polarity using phage λ and the λdv plasmid as DNA substrates recombined *via* the *E*. *coli* RecBCD system *in vivo*. These DNAs are mutant for λ recombination functions, including *orf* and *rap*, which were functional in previous studies. Heteroduplexes are isolated, separated on polyacrylamide gels, and quantified using Southern blots for heteroduplex analysis. This method reveals that heteroduplexes are still found in either 5′ or 3′ DNA strands in approximately equal amounts, even in the absence of *orf* and *rap*. Also observed is an independence of the RuvC Holliday-junction endonuclease on patch formation, and a slight but statistically significant alteration of patch polarity by *recD* mutation.

**Conclusions/Significance:**

These results indicate that *orf* and *rap* did not contribute to the presence of patches, and imply that patches occurring in both DNA strands reflects the molecular mechanism of recombination in *E*. *coli*. Most importantly, the lack of a requirement for RuvC implies that endonucleolytic resolution of Holliday junctions is not necessary for heteroduplex-patch formation, contrary to predictions of all of the major previous models. This implies that patches are not an alternative resolution of the same intermediate that produces splices, and do not bear on models for splice formation. We consider two mechanisms that use DNA replication instead of endonucleolytic resolution for formation of heteroduplex patches in either DNA strand: synthesis-dependent-strand annealing and a strand-assimilation mechanism.

## Introduction

**Figure 1:**
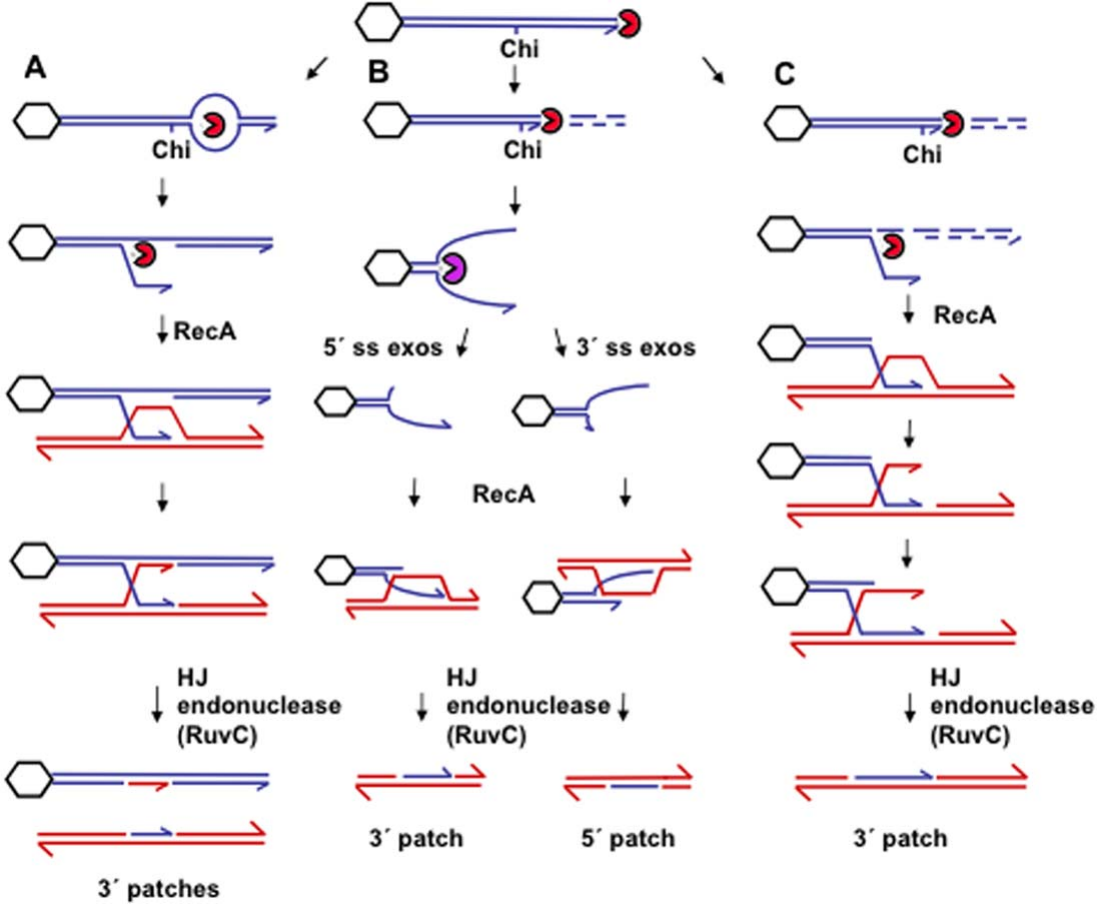
Previous models for RecBCD-mediated recombination in *E. coli.* RecBCD (notched circle) loads onto a double-strand end (DSE), depicted here as the right end of the phage λ chromosome, the left end being occluded during packaging by terminase and the packaging proteins (depicted as an octagon) [101]. (A) The nick-at Chi model [6], [7] suggested that RecBCD unwinds and rewinds the DNA until it encounters Chi, at which point it nicks the 3′-ending strand which invades a homologous duplex DNA molecule (red), creating patches exclusively in the 3′-ending strand. (B) The split-end model [21] proposed that RecBCD degrades both strands until an encounter with Chi, effectively translocating the DSE to the Chi site. At Chi, RecBCD was proposed to lose its nuclease activity, retain helicase activity, and unwind the DNA. This split-end intermediate might be acted on by single-strand-dependent exonucleases of one polarity or the other, creating single-strand ends of either polarity that could invade a homologous duplex DNA molecule [12]. 5′-end invasions were proposed to lead to 5′ patches, and 3′-end invasions to lead to 3′ patches. (C) The asymmetric DNA degradation model [8] incorporates the proposal of [21] that RecBCD degrades one or both (depicted here) DNA strands until an encounter with Chi, at which point this model specifies that the nuclease activity is altered and only the 5′-ending strand is degraded. This creates a 3′ end that invades a homologous duplex DNA molecule leading to patches exclusively in the 3′-ending strand. All of these models include endonucleolytic resolution of the strand-exchange intermediate, such as a Holliday junction (HJ), as the final step, a prediction that has been upheld for “break-join” splices [77], [78], but that will be called into question for patch formation by data presented below. We will suggest that none of these models can explain patch formation and consider alternatives. DNA ends with a half arrowhead represent 3′ ends, and plain ends represent 5′ ends.

RecBCD is a powerful exonuclease, and an important enzyme in homologous recombination in *Escherichia coli* [1]–[3]. In many models for RecBCD-mediated recombination *in vivo*, the enzyme is proposed to bind a double-strand DNA end (DSE) at a double-strand break [4], [5], and create single-stranded DNA with a 3′ end that is coated with RecA protein and invades a homologous duplex DNA molecule. The 3′ end has been hypothesized to be created in different ways. In one model, a single-strand nick is made in the strand ending 3′ (relative to the DSE where RecBCD loaded) when RecBCD encounters a Chi sequence in the proper orientation, from the 3′ side of 5′-GCTGGTGG [6], [7] (Figure 1A). Another model creates a single-stranded 3′ end by preferential degradation of the complementary 5′-ending strand by RecBCD after an encounter with Chi [8] (Figure 1C). Neither model accounted adequately for the strand polarity of heteroduplex DNA patches formed by RecBCD-mediated Chi-stimulated recombination *in vivo* [9]–[11]. Heteroduplex patches are a stretch of duplex containing strands from different parental DNA molecules. Both of these models assume that patches result from an alternative resolution of the same intermediate(s) that produce splices, and both predict that patches will contain donated DNA only in the 3′-ending strand, with respect to the DSE where RecBCD loaded.

However, *in vivo* and biochemical evidence support the presence of heteroduplex DNA in either 5′- or 3′-ending strands. First, RecBCD-mediated Chi-stimulated genetic [9], [10], then physical [11] analyses of heteroduplex patches formed *in vivo* into phage λ or plasmid DNAs showed patches in either strand of DNA. In these studies, RecBCD could load on only one end of the λ molecule that initiated recombination. Thus, the data suggested that either the 5′- or 3′-ending strand released by RecBCD upon end processing could initiate recombination that forms a patch *in vivo*. Second, *in vivo* experiments testing the role of single-strand DNA exonucleases in recombination showed a significant decrease in splice formation only when exonucleases of both polarities were removed [12], [13]. This suggested that single-strand exonucleases could create a recombination intermediate that contained an overhang of one polarity or the other, either of which could be used as a strand-exchange intermediate for recombination. Third, biochemical studies of DNA strand invasion with RecA protein suggest that 5′ or 3′ ssDNA ends are able to invade and form D-loops with a homologous duplex DNA *in vitro* [14], [15]. RecA polymerizes in a 5′ to 3′ direction on single-stranded DNA [16], making it obvious how 3′ ends can be coated, and perhaps accounting for preferential 3′-end invasion activity with purified RecA [17], [18]. However, when RecOR accessory proteins are added, 5′ ends can also be used in strand-exchange reactions [19], [20], perhaps accounting for the *in vivo* results.

These data appeared to be inconsistent with the recombination models with exclusively 3′-end invasions, and supported an alternative model [21]. The split-end model (Figure 1B), was the first to suggest that RecBCD loads onto DNA ends in its exonuclease mode and degrades/removes both strands of DNA until it encounters a properly oriented Chi site, effectively translocating DSEs to Chi. Upon an encounter with Chi, RecBCD was proposed to lose its exonuclease activity, but retain helicase activity. The helicase activity was hypothesized to be the recombinagenic activity of the enzyme, unwinding the DNA and creating single-stranded DNA ends of both polarities that are coated with RecA and capable of invading a homologous duplex DNA molecule. These ends might be subject to digestion by 5′ or 3′ single-strand exonucleases to create 5′ or 3′ overhangs (in different molecules, Figure 1B) [12], [13], which might then lead to patches of either polarity. Supporting this possibility, *either* 5′ *or* 3′ single-strand exonucleases are required for efficient Chi-stimulated recombination (splicing) of phage λ DNAs *in vivo*, implying that digestion of a strand of *either* polarity is required at some stage of the reaction *in vivo*, for example, to create a recombinagenic DNA end [12] (other interpretations are discussed below). Note that this, and the two other models described above (Figure 1A–C), all share the feature that patches are presumed to result from endonucleotylic cleavage of Holliday junctions, as an alternative product to splices—an idea that will be questioned below.

Analysis of the structures of heteroduplexes has been used to examine recombination products in order to probe the mechanism of recombination *in vivo*. Purely genetic methods used the *P* or *c*I genes of phage λ, and selection or screening for recombinant phage that had once contained heteroduplexes [9], [22]–[24], and also in yeast using the *his4* locus and tetrad analysis [25]. Hagemann and Rosenberg [11] examined the strand polarity of heteroduplex-patch recombinants physically using partially denaturing gel electrophoresis and found both 5′ and 3′ patches to be prevalent, with a small (two-fold) bias toward 5′ patches. Their heteroduplexes consisted of five mispairs within an 18 bp insert in the λ *ren* gene. Although reproducible, the method used was arduous and complicated. In addition, since that work, two open reading frames were identified in λ, *orf* and *rap*, that encode functions that might have influenced their results: Orf encodes an analog of RecO, RecR, and RecF strand-exchange accessory proteins [26]. Rap encodes a Holliday-junction and D-loop endonuclease [27].

This paper describes a different physical method for analysis of heteroduplex structure in patch recombinants formed *in vivo*. Heteroduplexes are formed *in vivo via* recombination of a λ phage that is wild-type at *ren*, and a λdv plasmid containing the 18 bp insert at *ren* [11] as recombination substrates. The 18 bp insert forms 18 nt looped heteroduplexes that can be separated from each other by native polyacrylamide gel electrophoresis (per the method of [28]). Separated DNA is then transferred from the gel to a membrane and hybridized with a general or specific non-radioactively labeled probe to identify and quantify heteroduplexes of each polarity (similar to [29]). The λ phage are mutant for their own recombination functions (*red* and *gam*), and allow RecBCD-mediated recombination ([30], and shown below).

λ chromosomes in cells are circular until packaging, at which point they acquire a DSB at *cos* with *one* DSE available for RecBCD loading: the phage λ chromosome right end only (as depicted in the standard λ map, and as shown at the top of Figure 1, reviewed [2]). The structures of heteroduplex-patch recombinants formed *in vivo* were re-examined, and compared with those from infections (crosses) using λ phage deleted for *orf* and *rap*. Patches formed in this system were found first to be dependent on RecA and RecB, as expected for *E*. *coli* RecBCD-mediated DSE-repair recombination. Second, although patch frequency is increased in the presence of functional λ *orf* and *rap* genes, approximately equal amounts of the two polarities of heteroduplex were obtained regardless of their presence, demonstrating no influence of λ Orf and Rap recombination proteins on patch-strand polarity, and implying that the patch polarity really does reflect *E*. *coli* recombination functions. Third, the loss of *recD* causes a small but statistically significant shift toward more 5′ patches. Fourth, surprisingly, RuvC, the Holliday-junction endonuclease, is not required for patching. This result suggests that none of the models shown in Figure 1, all of which require endonucleolytic cleavage of a Holliday-junction, bear on patch formation, though any of them might explain the formation of splices. We consider two different models in which patches form by a different route from that generating splices, and which uses DNA replication, rather than Holliday-junction cleavage, to resolve (non-Holliday-junction) intermediates.

## Materials and Methods

### Construction of strains, plasmids, and new alleles

Bacterial strains used in this study are given in Table 1. Plasmids pLGR4 and pLGR5, containing the lambda *orf* deletion (Δ*orf432*), a net deletion of 432 bp, were constructed by PCR using outward-facing primers Δorf-146 forward 5′-GGGTTT**GCTAGC**CTGATGCATCAGTTCGGGCTGCATGATAAATGTCG-3′ and Δorf-146 reverse 5′-GGGTTT**GCTAGC** AAGGCCTGCGATTACCAGC-3′ (underlined sequence has homology to the plasmid, and sequence in bold is the recognition site for *Nhe*I) on either side of the lambda *orf* gene present in the λdv derivatives pKC31 (made by R. N. Rao, Eli Lilly & Co., Indianapolis, IN, and is similar to pRLM4 [31], see also [32]) and pAH15 [11] respectively. (pAH15 is derived from pKC31 and contains an 18 bp insert including a *Xho*I restriction site [11].) This deletes λ base pairs 40,621–41,073, and inserts 21 bp including an *Nhe*I restriction site to create a net deletion of 432 bp. PCR products were digested with *Nhe*I (New England BioLabs), diluted, and ligated to form circular plasmids in which *orf* was deleted. Lambda strains (Table 2) were deleted first for *rap* (Δ*rap514*), a net deletion of 514 bp, by homologous linear replacement of the gene in λOH3 (*red3 gam210 imm^21^cts* SR4° χ^+^D *Sam7*) as a prophage. Primers Δrap-CAT forward 5′-TGATGAGCGATCCGAATAGCTCGATGCACGAGGAAGAAGATGATGGCTAAGTGTAGGCTGGAGCTGCTTC-3′ and Δrap-CAT reverse 5′-GTATGCTTCAACGAGCATGTCTGGAATGGTTTTTACTGAGAACGTCATGCCATATGAATATCCTCCTTAGT-3′ (underlined sequence has homology to the plasmid pKD3, and non-underlined sequence has homology to either side of the lambda *rap* gene) were used to amplify the chloramphenicol-resistance (*cat*) gene from pKD3 *per* [33]. The PCR product was transformed into strain AH9, which also contained pKD46 [33], and chloramphenicol-resistant transformants were selected and verified by PCR using primers nin5L 40.410 5′-GTCTTCTGGTTATCGAAGG-3′ and nin5R 43.522 5′-CTTTGTCGTAATCGAGATT-3′ to create strain SMR6230. The *cat* gene was removed from the prophage in SMR6230 using pFT-A to express FLP [34] to create strain SMR10233, and the *rap* deletion was verified by PCR using the same primers as above. This deletes λ base pairs 42,440–43,037, and inserts 84 bp including the remaining FRT “scar” to create a net deletion of 514 bp. The *rap* deleted prophage was then heat induced by shaking vigorously at 44°C for 15 minutes, followed by 37°C for 2 hours. Phage were isolated by lysing the culture, and were then infected into cells carrying pSR1 (λdv *imm^434^ Ots28* Kan^R^). Phage λ *Ots28* recombinants were screened for temperature sensitivity at 42°C, and then allowed to recombine with either pLGR5 or pLGR4 (which are *O* ^+^) and *O* ^+^ recombinants were selected at 42°C to create λ strains deleted for both *orf* and *rap*, with or without the 18bp insert (strains λSR543 and λSR542, respectively). The double deletion was verified by PCR using primers nin5R 43.522 and nin5L 40.410 (above). Stocks of λ phage were made by standard methods [35]. *E*. *coli* strains were made by standard P1 transduction methods [36].

**Table 1:** *E. coli* K-12 strains and plasmids

Strain/Plasmid	Relevant genotype	Source or reference
594^1^	Su^−^	[103]
AH9^2^	C600 *recD1009* (λ *red3 gam210 imm^21^cts* SR4° χ^+^D *Sam7*)	Ann Hagemann
DH5α	*endA1 hsdR17*(*r_K_* ^−^ *m_K_* ^+^) *supE44 thi-1 recA1 gyrA*(Nal^R^) *relA1 deoR* Δ(*lacZYA-argF*)*U169* (φ80 *dlac*Δ(lacZ)*M15*) λ^−^ F^−^	[104]
DPB271	*recD1903*::mini-*tet*	[87]
FS1607	594[pKC31]	F.W. Stahl, U. of Oregon
GY8322	Δ(*srlR-recA*)*306*::Tn*10* [mini-F K5353 (*recA* ^+^)]	S. Sommer, Gif sur Yvette, France
JC11450^3^	AB1157 Su^−^	A. J. Clark, U. of Arizona
JW1752	Δ*topB*::FRT-*kan*-FRT	C. Herman, Baylor College of Medicine, [105]
JW1852	Δ*ruvC*::FRT-*kan*-FRT	C. Herman, Baylor College of Medicine, [105]
SMR423	C600 SuIII^+^ *recD1903*::mini-*tet hsdr_K_^−^m_K_^+^*	[106]
SMR580	*recB21 argA*::Tn*10*	[107]
SMR2595	JC11450 *recB21 argA*::Tn*10*	JC11450×P1(SMR580)
SMR6205	594[pLGR4]	594 transformed with a ligation mix of *Nhe*I cut PCR fragment of pKC31 creating Δ*orf432*
SMR6230	C600 *recD1009* (λ *red3 gam210 imm^21^cts* SR4° Δ*rap*::FRT-CAT-FRT χ^+^D *Sam7*)	AH9 transformed with a PCR fragment containing the *cat* gene from pKD3 and homology flanking λ *rap*
SMR6668	DH5α[pLGR5]	DH5α transformed with a ligation mix of *Nhe*I cut PCR fragment of pAH15 creating Δ*orf432*
SMR6720	594[pLGR5]	594 transformed with pLGR5
SMR6721	594[pAH15]	594 transformed with pAH15
SMR6726	JC11450[pLGR5]	JC11450 transformed with pLGR5
SMR9579	JC11450 *recB21 argA*::Tn*10* [pLGR5]	SMR2595 transformed with pLGR5
SMR10152	JC11450 Δ(*srlR-recA*)*306*::Tn*10*	JC11450×P1(GY8322)
SMR10154	JC11450 Δ(*srlR-recA*)*306*::Tn*10* [pLGR5]	SMR10152 transformed with pLGR5
SMR10190	JC11450 *recD1903*::mini-*tet*	JC11450×P1(DPB271)
SMR10203	JC11450 Δ*topB*::FRT-*kan*-FRT	JC11450×P1(JW1752)
SMR10205	JC11450 Δ*topB*::FRT	SMR10203 transformed with pCP20, heat induced at 42°C, and screened for Kan^S^, Amp^S^
SMR10207	JC11450 Δ*topB*::FRT [pLGR5]	SMR10205 transformed with pLGR5
SMR10210	JC11450 Δ*ruvC*::FRT-*kan*-FRT	JC11450×P1(JW1852)
SMR10211	JC11450 Δ*ruvC*::FRT	SMR10210 transformed with pCP20, heat induced at 42°C, and screened for Kan^S^, Amp^S^
SMR10213	JC11450 Δ*ruvC*::FRT [pLGR5]	SMR10211 transformed with pLGR5
SMR10215	JC11450 *recD1903*::mini-*tet* [pLGR5]	SMR10190 transformed with pLGR5
SMR10233	C600 *recD1009* (λ *red3 gam210 imm^21^cts* SR4° Δ*rap514* χ^+^D *Sam7*)	SMR6230 transformed with pFT-A which expresses FLP to remove the *cat* gene
pAH15	derivative of pKC31, with *Xho*I^40.4^: an 18 bp insert that contains a *Xho*I site in the *Sst*II site at *ren*, Kan^R^	[11]
pCP20	Expresses FLP by heat induction at 42°C, Amp^R^, Cam^R^, Temp^S^ (≤30°)	[33]
pFT-A	Expresses FLP site specific recombinase by induction with Chlortetracycline, Amp^R^	[34]
pLGR4	Derivative of pKC31 with the λ *orf* gene deleted, Kan^R^	This work.
pLGR5	Derivative of pAH15 with the λ *orf* gene deleted, Kan^R^	This work.
pKC31	Derivative of λdv, contains the *Hind*III to *BamH*I fragment of phage λ including from the end of *c*I to the beginning of *orf-290*, Kan^R^	R. N. Rao [31], [75]
pSR1	λdv *imm^434^ Ots28* Kan^R^	S.M. Rosenberg and F.W. Stahl, U. of Oregon

Abbreviations: Amp^R^, ampicillin resistant; Kan^R^, kanamycin resistant; Cam^R^, chloramphenicol resistant; Temp^S^, temperature sensitive for growth. The Δ*rap514* and Δ*orf432* alleles are deletions of λ base pairs 42,440–43,037 and 40,621–41,073, respectively.

Footnotes: ^1^ Other genetic elements present in 594: *galK2*(*Oc*), *galT22*, *lac-3350*, *rpsL179*, *IN*(*rrnD-rrnE*)*1*.

2 Other genetic elements present in the C600 strain background: *supE*, *thi-1*, *thr-1*, *leuB6*, *lacY1*.

3 Other genetic elements present in JC11450: *thr-1*, *leuB6*, Δ(*gpt-proA*)*62*, *hisG4*, *argE3*, *thi-1*, *ara-14*, *lacY1*, *galK2*, *xyl-5*, *mtl-1*, *tsx-33*, *rpsL31*, *kdgK51*.

**Table 2:** λ phage strains

Strain	Genotype	Source or reference
λOH3	*red3 gam210 imm^21^cts* SR4° χ^+^D *Sam7*	Ann Hagemann
λSR537	*red3 gam210 imm^21^cts Xho*I^40.4^ χ^+^D *Sam7*	λOH12 [11], induced by Ann Hagemann
λSR538	*red3 gam210 imm^21^cts Xho*I^40.4^ χ^+^D *Sam7*	λOH12, grown from λSR537
λSR539	*red3 gam210 imm^21^cts* SR4° χ^+^D *Sam7*	λOH3, heat induced from AH9
λSR540	*red3 gam210 imm^21^cts* SR4° Δ*rap514* χ^+^D *Sam7*	Heat induced from SMR10233
λSR541	*red3 gam210 imm^21^cts Ots28* SR4° Δ*rap514* χ^+^D *Sam7*	λSR540×pSR1
λSR542	*red3 gam210 imm^21^cts* SR4° Δ*orf432* Δ*rap514* χ^+^D *Sam7*	λSR541×pLGR4
λSR543	*red3 gam210 imm^21^cts* SR4° *Xho*I^40.4^ Δ*orf432* Δ*rap514* χ^+^D *Sam7*	λSR541×pLGR5

Δ*rap514* and Δ*orf432* are deletions of λ base pairs 42,440–43,037 and 40,621–41,073, respectively (Materials and Methods). *red3*, an unconditional *red* ^−^ allele [108]; *gam210*, an amber allele suppressible by *E*. *coli* SuII^+^ or SuIII^+^ [109]; *imm^21^cts*, phage 21 immunity region substituted into λ with a temperature-sensitive clear-plaque mutation [110]; SR4°, mutation inactivating the λ *Eco*RI 4 site [111]; χ^+^D, Chi^+^ mutation between the λ *S* and *R* genes [112]; *Sam7*, SuIII^+^-suppressible amber mutation in the essential *S* gene [113]; *Xho*I^40.4^, 18bp insertion containing a *Xho*I site in the *ren* gene [11].

### λ by λdv crosses

Bacterial strains carrying an *orf* deleted plasmid, pLGR4 or pLGR5, were inoculated into Mating Culture Broth (MCB = LBK [37], plus 5mM MgSO_4_, 10mM Thymine, and 10mM Vitamin B1) at a dilution of 1∶100 from saturated overnight cultures grown in LBK with 50 µg/mL kanamycin shaking at 37°C. Mating cultures (enough for 5.0–10.0 mL) were grown for at least 2.5 hours at 37°C in the presence of 50 µg/mL kanamycin until a density of 1.5×10^8^ cells/mL was reached, as determined using a Petroff-Hausser counter. Phage were introduced at a multiplicity of infection of 7, and allowed to infect cultures for 30 minutes at 37°C while shaking. 25mL of cold TM [38] buffer was added to the infected cells, which were then pelleted and resuspended in 50 or 100 mL of prewarmed MCB with kanamycin, and allowed to shake vigorously (300 rpm) for 3–4 hours at 37°C. Cultures were then centrifuged for 10 minutes at 7,000 rpm and pellets were stored at −20°C until recombinant plasmid was isolated.

### Isolation of DNA

Plasmid DNA from crosses was isolated, and purified over CsCl gradients as described [38]. Ethidium bromide was extracted using isoamyl alcohol [38], and CsCl was removed by dialysis against TE [38] using Slide-A-Lyzer Dialysis Cassettes, 10,000 MWCO (Pierce, Rockford, IL). Plasmid DNA preparations were then ethanol precipitated, and the pellets were dissolved in ddH_2_0. Any remaining lambda DNA was removed by linearization with *Ase*I (New England BioLabs), which does not linearize any of the plasmids used here, but cleaves λ; followed by digestion with Plasmid-Safe ATP-Dependent DNase (EPICENTRE, Madison, WI) overnight at 37°C. Pure plasmid DNA was again ethanol precipitated, and the pellets dissolved in 10 mM Tris pH 8.0.

### Analysis of recombinants

The yield of plasmid DNA isolated from crosses was checked by agarose gel electrophoresis in 1X TAE buffer [38]. The amount of DNA to be run on an acrylamide gel was determined empirically because the plasmid DNA contained multimers and therefore could not be compared directly to a DNA size marker for estimation. (DNA concentration could not be determined using a spectrophotometer due to the low yields obtained from crosses.) DNA was digested with *Stu*I (New England BioLabs) to release a 604 bp fragment containing the loop heteroduplex. Artificial heteroduplexes were prepared by digesting pLGR4 (no insert) and pLGR5 (with the 18 bp insert) with *Stu*I, mixing equal amounts of each digest, boiling the mixture for 10 minutes, and allowing the duplexes to reanneal at 65°C for 20 minutes. Either one or two different amounts of DNA for each cross was loaded onto a 5% native polyacrylamide gel (BioRad), and run in 1X TBE buffer (BioRad) at room temperature for approximately 140 minutes at 110V. (Undiluted restriction digests were loaded using Ficoll loading dye, diluted digests were loaded using glycerol loading dye [39].) After electrophoresis, the DNA in the gel was denatured for 20 minutes in denaturation buffer (0.5 M NaOH, 1.5 M NaCl [38]), and neutralized in 1X TBE 3 times for 20 minutes each (George Church, personal communication). DNA was electroblotted to a positively charged nylon membrane (Roche) in very cold 1X TBE using an Electrophoretic Mini-Blotter (C.B.S. Scientific) at 75V for 2.5–3 hours (or 70V for 3–4 hours) with constant voltage in the cold room. After transfer, the DNA was fixed onto the blot by UV crosslinking with 1600 J/m^2^ [40] and the blot was stored at 4°C.

Blots were hybridized with either digoxygenin (DIG)-labeled oligonucleotide probes specific for the two complementary heteroduplex loops, or with a 700 bp DIG-labeled PCR product homologous to the restriction fragment containing the heteroduplex (similar to [29]). The two 18 nt oligonucleotide probes; 5′ probe–*Xho*I40.4 R strand probe 5′-AGGGCTCGAGCGGGGAGC-3′, 3′ probe–*Xho*I40.4 L strand probe 5′-GCTCCCCGCTCGAGCCCT-3′; were labeled by creating a tail of DIG molecules on the 3′ end of each using the DIG Oligonucleotide Tailing Kit (Roche). These were hybridized to blots in sequential hybridization reactions using an aqueous hybridization buffer similar to that suggested by Roche [5X SSC, 2% Blocking Reagent (Roche), 0.1% N-Lauroyl Sarcosine, 1% SDS, and 0.1 mg/mL polyA (Roche)] at 47.5°C overnight with 0.15 pmol/mL oligonucleotide probe. Blots were washed twice for 5 minutes each with 2X SSC, 0.1% SDS at room temperature; and then three times for 10 minutes each with 0.5X SSC, 0.1% SDS at 60°C. The PCR probe was labeled using the PCR DIG Probe Synthesis Kit (Roche), with primers *ren* Left 40.232 5′-GAAGATCGCAGAAATCAAA-3′ and *ren* Right 41.364 5′-ATAAATGGCTTCAGAACAG-3′ and hybridized to blots using DIG EasyHyb Buffer (Roche) with 0.1 mg/mL sheared salmon sperm DNA (Ambion) at 65°C overnight with 10 µL probe. Blots were washed twice for 5 minutes each at room temperature as above, and then three times for 10 minutes each with 0.1X SSC, 0.1% SDS at 72°C. All blots were then developed for quantification of heteroduplex bands using CDP-Star substrate (Roche) according to kit protocol. Developed blots were either exposed to XAR5 film (Kodak) for between 10 seconds and 2 hours, depending on which probes were used, or imaged with a ChemiImager 5500 (Alpha Innotech, San Leandro, CA). Images were acquired in 5 minutes or less, and were quantified using AlphaEase software (Alpha Innotech). This method has been shown to be quantitative [41]–[43]. Blots that were probed multiple times were stripped according to kit protocol. Oligonucleotide probes were stripped at 50°C, and PCR probes at 65°C.

### Analysis of λ patch recombinant frequencies

Recombinant frequencies were determined by diluting and plating phage progeny from crosses on *E. coli* indicator strain SMR423 (Table 1) for 100–1000 plaques per plate/blot. Plaque blots were made on positively charged nylon membranes (Roche) and processed according to manufacturer's instructions. Blots were hybridized with DIG-labeled oligonucleotide probes as above, except that both probes were hybridized to blots together in the same hybridization solution instead of separately. Hybridized blots were developed as above, and exposed to XAR5 film (Kodak) for 5–20 minutes.

## Results

### Complementary heteroduplexes can be separated and identified

**Figure 2:**
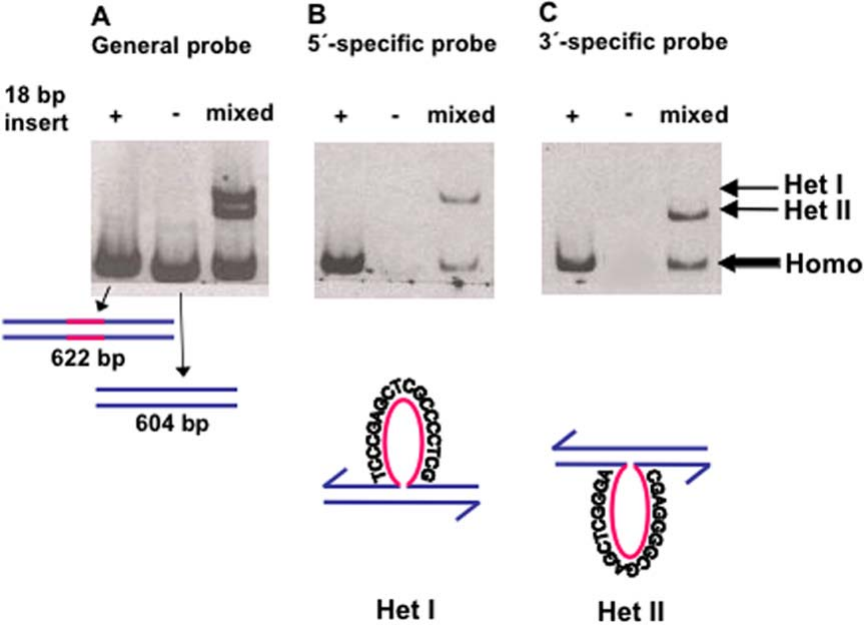
Separation of heteroduplexes by polyacrylamide gel electrophoresis. Southern blot of artificial heteroduplexes, made by melting and reannealing the 604 bp long DNA fragment with and without an 18 bp insertion marker, run on a 5% polyacrylamide gel. (A) Hybridization of the blot with a PCR-labeled probe complementary to the entire 604 bp restriction fragment. (B) Hybridization with an oligo probe complementary to the loop sequence in the strand ending 5′ at the right. (C) Hybridization with an oligo probe complementary to the loop sequence in the 3′-ending strand. “+”, the homoduplex fragment containing the 18 bp insert; “-”, the homoduplex fragment with no insert; “mixed”, melted and reannealed “+” and “−” DNAs (artificially prepared heteroduplexes). Figures beneath the gels represent the structures of Homo- and Het-containing fragments, showing the sequences of the complementary loops. “Het”, heteroduplex; “Homo”, homoduplex.

The heteroduplexes examined in this study consist of an 18 nt loop in one or the other strand of a 604 bp restriction fragment. These heteroduplexes are products of recombination between a λ phage (mutant for its recombination functions) and a λdv-derived plasmid. Heteroduplex recombinants containing at least 19 nt heteroduplex loops have been observed in λ recombination [44], and such heteroduplexes are not repaired in *E. coli* [44]–[46]. The λdv-derived plasmid pLGR5 contains approximately 4 kb of homology to the λ phage, and an 18 bp heterologous insert [11]. Because the two possible heteroduplex species have single-strand loops with unique complementary sequences (Figure 2), they are chemically distinct, and therefore should be capable of being separated from each other and from their respective homoduplexes [28].

**Figure 3:**
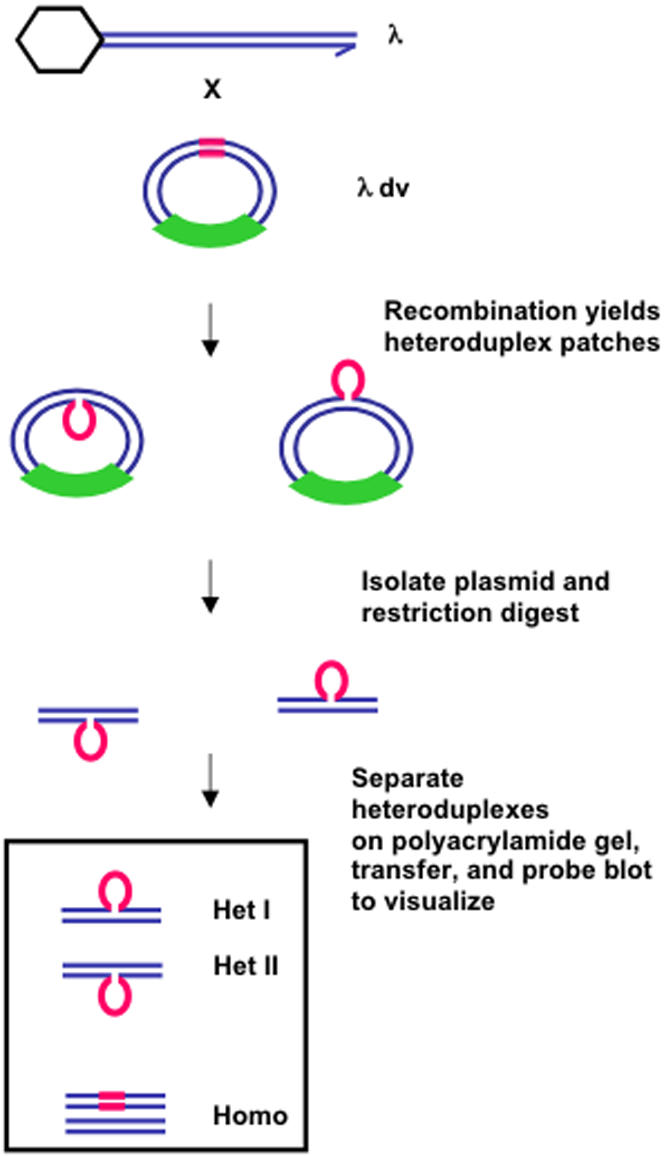
Strategy for heteroduplex analysis of patches formed *in vivo* in λ by λdv “crosses”. Red, 18 nt insertion marker; green box, region of the λdv plasmid DNA that is not homologous with λ; blue parallel lines, strands of DNA; hexagon, phage λ capsid and packaging proteins which bind the λ chromosome left end during cleavage of λ DNA for packaging. This leaves only the right end free for RecBCD-initiated recombination [102].

**Figure 4:**
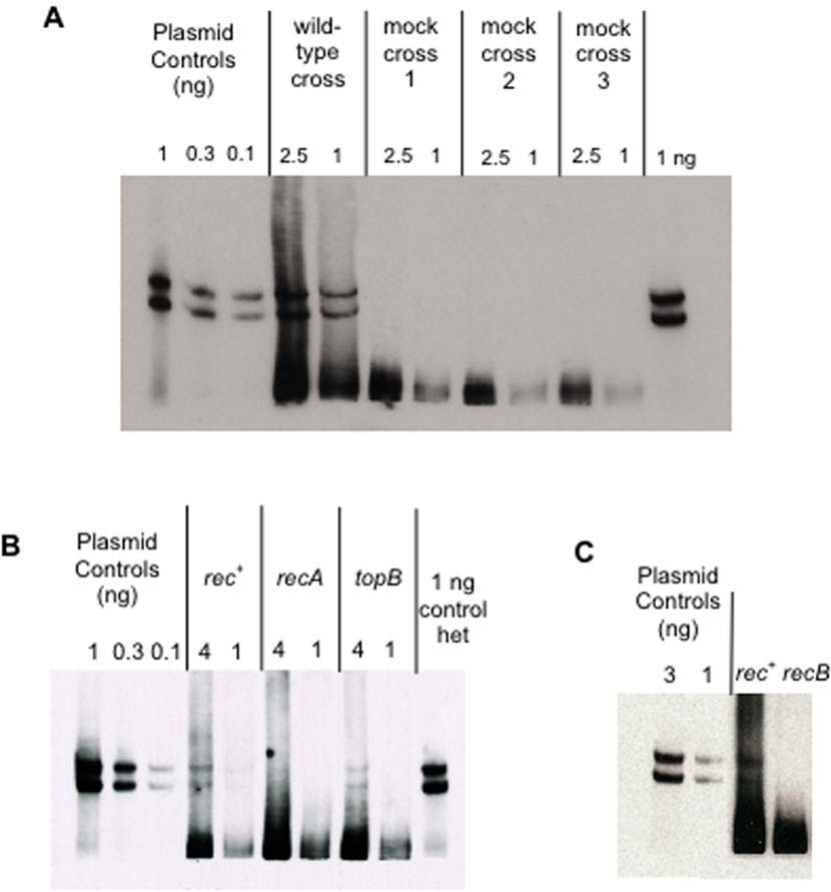
Heteroduplex patches are formed *in vivo*, RecA- and RecB-dependently. (A) Heteroduplexes are formed *in vivo*. Lanes labeled “wild-type” are two different amounts of DNA from a *rec* ^+^ cross (SMR6726×λSR542), lanes labeled “mock cross 1–3” contain two different amounts of DNA from each of three mock crosses (JC11450×λSR542 mixed with SMR6726 without λ infection, as described in text). The last lane also contains an artificial het control. (B) RecA dependence of het patches. Strains used are: *rec* ^+^ (SMR6726–JC11450 [pLGR5]), *recA* (SMR10154-JC11450 Δ(*srlR-recA*)*306*::Tn*10* [pLGR5]), and *topB* (SMR10207-JC11450 Δ*topB*::FRT [pLGR5]) each crossed with λSR542. Numbers above the lanes indicate the relative amounts of DNA loaded (*i. e*. a lane marked “4” indicates that lane contains four times the amount of DNA than was loaded in the lane marked “1” for the same cross). No het bands are visible for *recA* on the exposed film, but the scan of the film gives the appearance of bands present. The last lane is an artificial het control. (C) RecB dependence of het patches. Strains used are: *rec* ^+^ as in B., and *recB* (SMR9579–JC11450 *recB21* [pLGR5]). Plasmid controls are artificial het controls as in Figure 2. The homoduplex band was run off of the gel for the control lanes, but because the homoduplex fragment was 50–100x more prevalent in the cross DNAs, the homoduplex band was much broader and the upper portion of that band remained visible.

**Figure 5:**
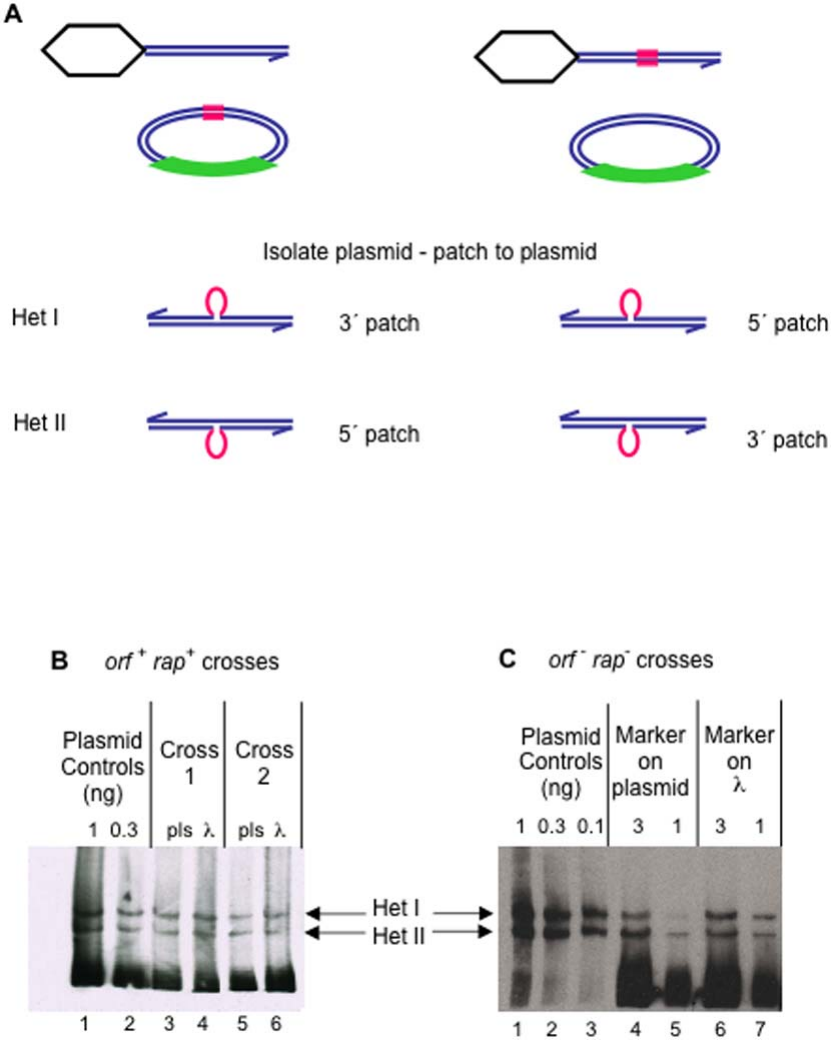
Prevalence of 5′- and 3′-strand patches is independent of λ Orf and Rap. (A) For analyses of plasmid DNAs isolated from crosses with the marker in the plasmid, Het I corresponds to a 3′ patch, and Het II corresponds to a 5′ patch. When the marker is in the λ, Het I corresponds to a 5′ patch and Het II to a 3′ patch. Red lines represent the 18 bp marker insert. (B) Plasmid DNAs isolated from *orf* ^+^ *rap* ^+^ crosses. Two crosses are shown. For each set of crosses, the lane marked “pls” denotes that the insertion marker was present in the plasmid (SMR6721×λSR539), and the lane marked “λ” denotes that the marker was present in the λ (FS1607×λSR538). See text for quantification. (C) Plasmid DNAs from *orf* ^−^ *rap* ^−^ crosses. One cross of each type is shown (marker present in the plasmid [SMR6720×λSR542] or in the λ [SMR6205×λSR543]), and two different amounts of DNA were loaded for each cross. Numbers above the lanes are relative amounts based on the estimated DNA concentration. Plasmid controls, artificial heteroduplexes made by melting and reannealing 604 bp fragments with and without the 18 bp marker insert as for Figure 2. See text for quantification from multiple crosses.

**Figure 6:**
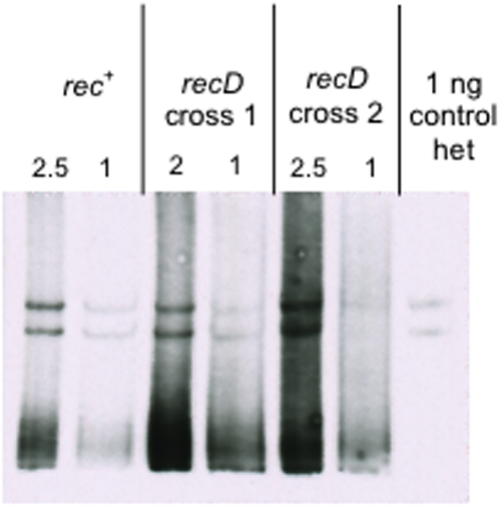
Crosses in a *recD* strain show a slight 5′ bias in patch polarity. Lanes marked “*rec* ^+^” are two different amounts of DNA from a *rec* ^+^ cross (SMR6726×λSR542). Lanes marked “*recD* cross 1” are two amounts of DNA from a *recD* cross (SMR10215×λSR542), and lanes marked “*recD* cross 2” are from a second *recD* cross. Control het is as for Fig. 2. For all crosses the 18 bp marker was present on the λdv plasmid, (SMR6726-JC11450 [pLGR5], SMR10215-JC11450 *recD1903*::mini-*tet* [pLGR5]).

**Figure 7:**
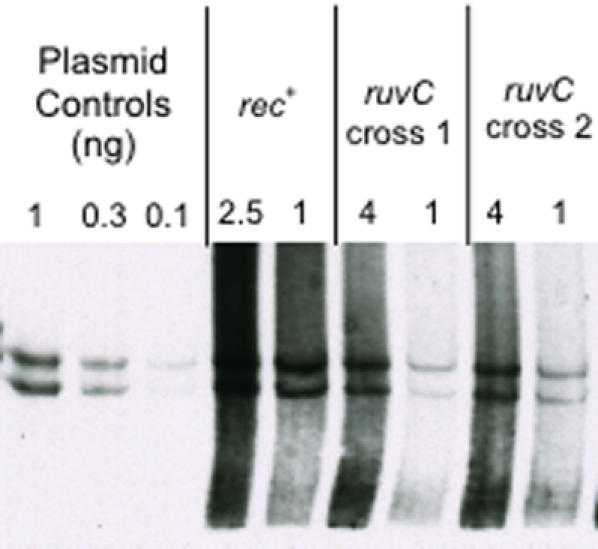
Loss of RuvC does not affect heteroduplex-patch formation. Strains are from left to right, *rec* ^+^ (SMR6726–JC11450 [pLGR5]), and both *ruvC* crosses were performed using (SMR10213–JC11450 Δ*ruvC*::FRT [pLGR5]), each infected with λSR542. See text for quantification.

**Figure 8:**
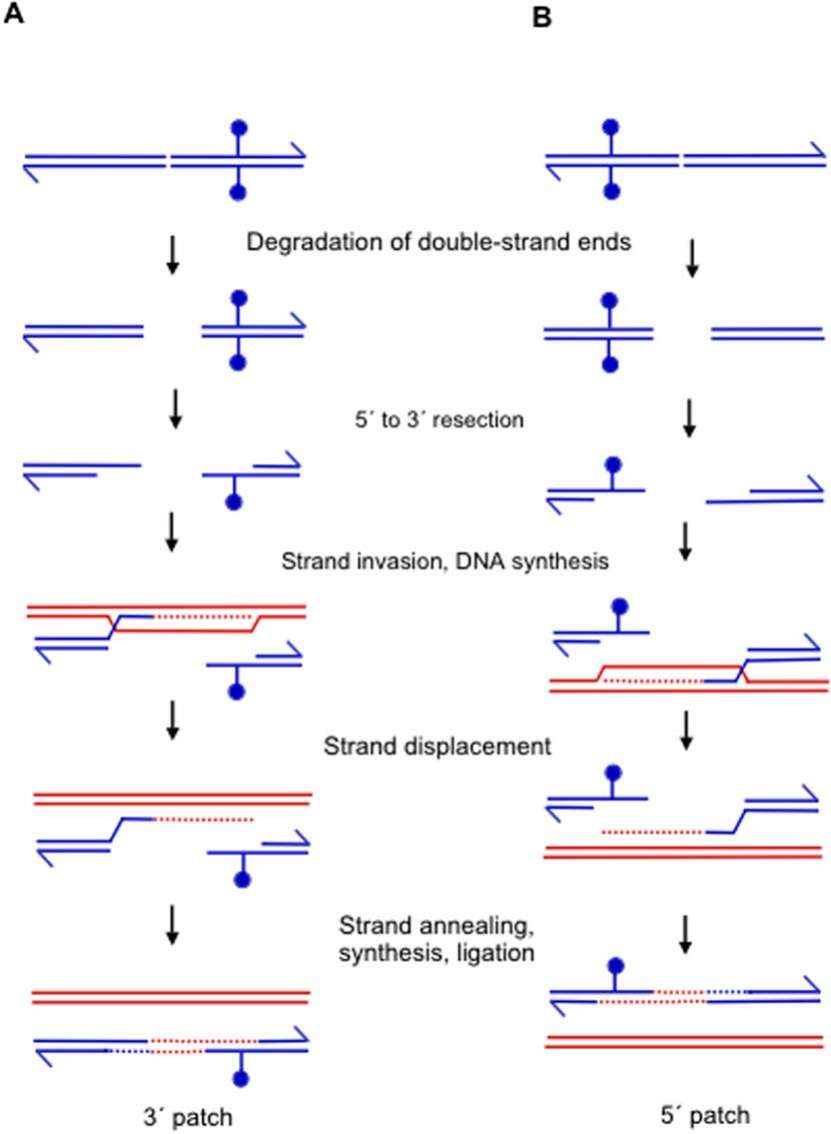
An SDSA model for the formation of 3′ and 5′ patches. (A) A DSB occurs to the left of the marker. 5′ ends are resected, and one of the resulting 3′ ends invades a homologous duplex. DNA synthesis (dotted lines), strand displacement, and reannealing lead to heteroduplex-patch formation with new DNA in the 3′-ending strand. Only the 3′ end that leads to heteroduplex formation across from the marker is shown. (B) A DSB occurs to the right of the marker. 5′ end resection, strand invasion, DNA synthesis, and reannealing all occur as in A, but result in heteroduplex patch formation with new DNA in the 5′-ending strand. Again, only the 3′ end that leads to heteroduplex formation across from the marker is shown. Adapted from Allers and Lichten [92].

**Figure 9:**
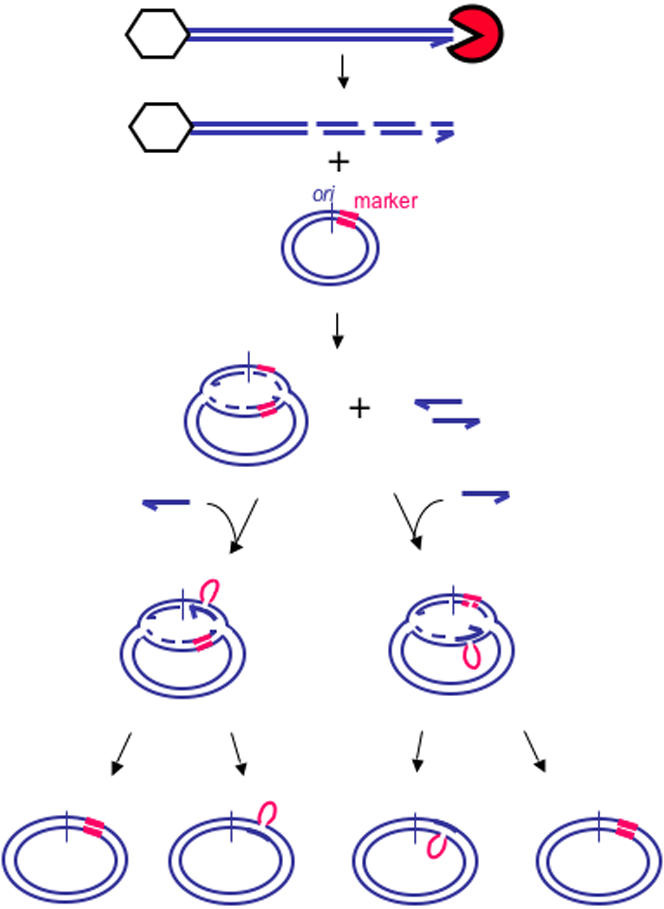
A single-strand-assimilation model for the formation of 5′ and 3′ patches. RecBCD loads onto λ at a DSE, and degrades both strands of DNA into ssDNA fragments. When these fragments are present in a cell at the same time as a replicating λdv plasmid (with which some fragments have homology) some of the fragments may be assimilated into the plasmid during replication. Fragments may also form RecA-dependent paranemic joints with the plasmid, as depicted in Leung *et al* [96] before replication begins. Once a replication fork passes over the region of the joint, the ssDNA fragment will be incorporated into the newly synthesized DNA strand. Fragments that are assimilated across from the 18 bp insert marker (which is not present in the λ phage) will result in a heteroduplex patch at that site. This assimilation might occur with ssDNA from either DNA strand of λ being assimilated across from its complementary DNA in either the leading or lagging strand of a replication fork, creating heteroduplex patches of either polarity (drawn). Adapted from Leung *et al.* [96] and Ellis *et al.* [97]; Court *et al.* [98]. Alternatively, assimilation might occur preferentially into the lagging strand, as for Red-mediated recombination [96]–[98] (not drawn), but because λdv has no replication terminus, replication might proceed in either direction *in vivo*, such that both strands are sometimes lagging strands. Thick blue lines represent DNA from the λ molecule, medium blue lines represent DNA from the λdv plasmid, thin blue lines represent newly synthesized λdv DNA, and pink lines represent the 18 bp insert marker. *ori* indicates the origin of replication for the λdv plasmid, but is not drawn to scale. RecBCD is depicted as in Figure 1, as a notched circle.

In Figure 2 we show that separation of these particular heteroduplexes can be achieved on polyacrylamide gels, and identified by Southern blotting, using artificial heteroduplexes made by melting and reannealing the DNAs with and without the insert. Figure 2A shows the migration of the two reannealed homoduplex restriction fragments, and the more slowly migrating heteroduplex species. The blot was then stripped and re-probed separately using oligonucleotide probes specific for the heteroduplex loop in either the 5′ or 3′ ending strand in each heteroduplex. Figure 2B shows that the most slowly migrating band contains the loop in the 5′ ending strand of the duplex (with respect to the right end of the fragment relative to the standard λ map), which will be referred to as heteroduplex (Het) I. Similarly, Figure 2C shows that the lower band of the two heteroduplex species (the middle band on the gel) contains the loop in the 3′ ending strand of the duplex, and will be referred to as Het II.

### Strategy for heteroduplex analysis *in vivo*

Heteroduplex patches were generated *in vivo* by infecting *E. coli* strains carrying a λdv-derived plasmid with phage λ that lacks λ recombination functions *red*, *gam*, *orf*, and *rap* (Figure 3). The λ and λdv were allowed to recombine, after which plasmid DNA was isolated and analyzed for recombinant patches in the region carrying the insertion marker. (During the 3–4 hour incubation step during which recombination was allowed, multiple recombination events may happen to a λdv molecule. Replication of a λdv molecule across a heteroduplex patch would erase that patch, however new recombination events with another λ molecule would restore the heteroduplex region. Therefore, allowing replication would not lead exclusively to homoduplex patches.) Plasmid DNA was restriction digested to release a 604 bp fragment containing the heteroduplex. Digests were run on polyacrylamide gels to separate heteroduplexes of each polarity from one another and from homoduplexes, transferred to membranes, hybridized with a non-radioactively labeled probe complementary to the restriction fragment, and bands visualized with a chemiluminescent substrate and quantified.

### Formation of 5′ and 3′ patches *in vivo*, RecA and RecB dependently

Several controls show that the heteroduplex patches observed here are formed *in vivo* and are products of RecBCD-mediated recombination. First, when λ is infected into a cell carrying λdv and the two molecules are allowed to recombine (referred to here as a cross), heteroduplexes of both polarities are obtained in an approximately 1∶1 ratio for 5′ to 3′ patches (Het II:Het I, Figure 4A). This appears similar to the 2∶1 ratio of 5′ to 3′ patches observed by Hagemann and Rosenberg [11].

Second, mock crosses show that the heteroduplexes tested in the experiments reported here formed *in vivo*, and not as an artifact of DNA isolation. The *rec* ^+^ strain carrying pLGR5 was treated as for a cross, but no λ phage were added to the culture. A similar *rec* ^+^ strain lacking the plasmid was infected with the same λ phage used in the above crosses, and the two cultures were incubated as for a cross. These cultures were then mixed together and plasmid DNA prepared as for crosses. Figure 4A shows that no heteroduplex species are detected in three mock crosses. Thus, the heteroduplexes observed in λ by λdv crosses were formed *in vivo*.

Third, λ by λdv crosses were performed in strains lacking either RecB, which harbors the nuclease domain of RecBCD [47], or RecA, the strand-exchange protein. Recombination of linear DNA is generally severely reduced in strains lacking either of these two proteins (reviewed by [1], [2], [48], [49]). Figures 4B and C reveal that no recombinant heteroduplex patches are observed in crosses performed in strains lacking either RecA or RecB, respectively, but are observed in *rec* ^+^ crosses. This reflects a >99% decrease in the number of patches to λdv observed in crosses performed in a strain lacking *recA* as compared to *rec* ^+^ on the blot, and a >90% decrease in patches observed in a strain lacking *recB* (ratio of signal in patch area to DNA input). Additionally, frequencies of patch recombinants from crosses in these strains were quantified by plaque-blot hybridization in which λ phage isolated from these crosses were hybridized with a probe to the marker in the λdv plasmid. Because splices of the λdv into the λ create a λ molecule too large to be packaged, examination of λ DNA with plaque blots measures RecBCD-mediated patches only. Table 3 shows that patches from λdv to λ are reduced as expected in strains lacking *recA* or *recB*. These data confirm that the heteroduplexes observed are the result of an *in vivo* RecABC-dependent recombination reaction.

**Table 3:** Patch frequencies measured by plaque hybridization

Strain	Relevant Genotype	λ Phage Titer	Patch Recombinant Frequency (%)^1^
SMR6726	*rec* ^+^	1.1×10^10^	1.4±0.9
SMR10154	Δ*recA*	2.8×10^8^	0.04±0.01
SMR9579	*recB21*	5.8×10^8^	0.12±0.09
SMR10213	Δ*ruvC*	3.5×10^9^	1.5±0.1
SMR10207	Δ*topB*	6.2×10^9^	2.2±1.1
λSR539	*orf* ^+^ *rap* ^+^	1.0×10^10^	12±1^2^
λSR542	*orf* ^−^ *rap* ^−^	1.6×10^10^	1.9±0.2^2^

1 Average and SD of three crosses.

2 Average and SE of three crosses, three blots each.

Frequencies of λ patch recombinants were assayed by plaque-blot hybridization (Materials and Methods). For the first five sets of crosses, λSR542 was infected into the bacterial strains shown. For the last two sets (which were performed separately from the first five), the λ strains indicated were infected into bacterial strains SMR6721 and SMR6720, respectively.

### λ Orf and Rap affect the frequency but not the polarity of heteroduplex patches

Previous examination of heteroduplex patches formed during RecBCD-mediated recombination of phage λ DNA revealed patches in either the 5′- or 3′-ending strand, (with respect to the right end of phage λ onto which RecBCD loads [11]), and showed a roughly two-fold bias toward patches in the 5′-ending strand (the λ *r* strand) [11]. Since that study, two new λ recombination proteins were discovered. The λ *orf* gene product (open reading frame previously called *orf-146*) is an analog of and can substitute for *E. coli* RecFOR proteins [26], [50]–[52], which assist RecA loading [53]. The λ *rap* gene product (open reading frame previously called *ninG*) encodes a Holliday-junction endonuclease that also cleaves D-loops [27], [54]–[56]. Its homolog, *E. coli* Rus, substitutes for the Holliday-junction-cleavage activity of the *E. coli* RuvABC system [57], and can also substitute for human WRN [58] and fission yeast Rqh1 [59] recombination proteins. Either Orf or Rap or both of these proteins could have influenced the formation of patches examined previously, either by promoting formation of 5′-strand invasions (Orf), or by biasing Holliday-junction cleavage in favor of 5′ patches (Rap).

To address whether the prevalence of 5′ and 3′ patches seen previously was influenced by these λ recombination proteins, patch-strand polarity was re-examined in their absence (see previous section and results to follow). Precise deletions of *orf* and *rap* were made in the λ phage and the λ-derived plasmids used here, and crosses of λ by λdv were conducted as previously [11]. Frequencies of patches from λdv into λ were determined in the presence and absence of Orf and Rap, *via* plaque-blot hybridization (Table 3). The recombinant frequencies from *orf* ^+^ *rap* ^+^ crosses were ∼six-fold higher than those from *orf* ^−^ *rap* ^−^ crosses (Table 3). This is comparable to a two-fold decrease in patch frequency measured previously in λ by plasmid crosses when the λ was *rap* ^−^ [60]. When recombinant frequencies of splices were measured in similar crosses, decreases of 17-fold [60] and 100-fold [56] were observed in *rap* ^−^ λ, suggesting that *rap* has a greater influence on splices than on patches. To analyze heteroduplex-patch-strand polarity, plasmids were purified from these mixed infections and assayed on polyacrylamide gels (Figure 5). In the first of these crosses, the marker insert was in the λdv plasmid, and recombinant plasmid was isolated, such that Het I represents patching of wild-type DNA from the λ phage into the plasmid 3′ strand, creating a 3′ patch with a loop in the 5′-ending strand of the restriction fragment (Figure 5A, left). Conversely, Het II represents patching of DNA from the λ phage into the plasmid 5′ strand, creating a 5′ patch with a loop in the 3′-ending strand of the restriction fragment.

The average of two crosses that were *orf* ^+^ *rap* ^+^, produced heteroduplex patches with a ratio of 5′ patches to 3′ patches of 1.0±0.2∶1 (average±range; no bias toward one or the other) (Figure 5B lanes 3 and 5). This result is roughly similar to the previous report from similar crosses, in which the bias toward 5′ patches was roughly two-fold [11]. In both studies, both 5′ and 3′ patches are prevalent. Crosses in which *orf* and *rap* were deleted also show roughly equal numbers of 5′ and 3′ patches, and might show a very slight bias toward 5′ patches; the 5′ to 3′ patch ratio was 1.1±0.3∶1 (average±S.D., n = 6) (Figure 5C lanes 4 and 5). These two ratios were not significantly different from each other (*P* = 0.874, by one way ANOVA).

Similar crosses were also performed with the marker in the λ instead of the λdv to ensure that any bias detected was not an artifact of marker placement. If some as yet undiscovered heteroduplex repair system in *E. coli* could repair 18 nt loops, and if it preferentially repaired either Het I or Het II, reversing the marker configuration would control for this by switching which patch type (*e.g.* 5′) is represented by which Het type (*e.g.* Het I), when the markers are reversed. In this configuration, Het I reflects 5′ patches of the marker insert from the λ into the plasmid, and Het II represents 3′ patches of the marker insert into the plasmid (Figure 5A, right). These *orf* ^−^ *rap* ^−^ crosses produced a 5′ to 3′ ratio of 0.9±0.1∶1 (or 1∶1.1, average±SD, n = 6) (Figure 5C lanes 6 and 7). Statistical analysis by one-way ANOVA indicated that the 1.1∶1 ratio when the marker was in the plasmid was not significantly different from the 0.9∶1 ratio when the marker was in the λ. That is, any possible bias was not strong enough to show a significant difference when the markers were swapped. For the *orf* ^+^ *rap* ^+^ crosses, the ratio was 1.0±0.1∶1 (average±range, n = 2) with the marker in the λ (Figure 5B lanes 4 and 6). A lack of significance is expected with an almost 1∶1 ratio. These results indicate that the roughly 1∶1 ratio is not caused by preferential repair of a particular Het type which would otherwise be much better represented due to a patch-strand bias. These results also indicate that although Orf and Rap increase patch frequency (Table 3), they do not contribute to the relative prevalence of both 5′ and 3′ patches.

### RecD influences patch-strand polarity

RecD is a subunit of the RecBCD enzyme that regulates recombination negatively, and is required for the strong exonuclease activity of RecBCD. Some models for recombination in *E. coli* depict RecBCD as preferentially degrading the 5′-ending strand after an encounter with Chi [8], [61], [62]. RecBC(D^−^) is almost completely devoid of the exonuclease activity of wild-type RecBCD *in* vitro and *in vivo* [3], [63]–[65], but possesses helicase activity [4], [66]–[68]. In addition, the crystal structure of RecBCD shows a 5′-ssDNA end passing through the RecD subunit and the 3′ strand through RecB [69]. This suggested that a strain mutant for RecD might show a bias toward 5′ patches because 5′-ssDNA ends might not be degraded. Crosses performed in a *recD* mutant strain showed a slight yet significant bias toward 5′ patches (Figure 6), with a 5′∶3′ patch ratio of 1.3±0.2∶1 (mean±SD of four crosses). This ratio is significantly different from that for the *rec* ^+^ controls run in parallel at 0.91±0.11∶1 (*P* = 0.006, by one-way ANOVA). These results indicate that changes in the ratio of 5′∶3′ patches could be detected using this assay, and also provide the first evidence of a cellular mutation that influences patch-strand polarity. The data further confirm that the patch-strand polarities reported are the result of recombination *in vivo via* the RecBCD-mediated DSBR pathway.

### Heteroduplex patches form independently of RuvC

Most models for RecBCD-mediated recombination, including all shown in Figure 1, depict a Holliday-junction intermediate that requires endonucleolytic resolution to form recombinant products, either patches or splices. In *E. coli*, RuvAB and RecG catalyze branch migration of Holliday junctions, and endonucleolytic resolution of the junction is performed by RuvC [70]–[73]. Models have been drawn that illustrate how the invasion of a 3′-ssDNA end could be resolved to form a heteroduplex on the 5′-ending strand, a 5′ patch, depending on the orientation in which RuvC resolves the junction [2], [9], [11], [22], [74]. There has also been speculation that *E. coli* topoisomerase III (TopB) might act to resolve Holliday junctions non-nucleolytically [75], and evidence that it acts in a pathway alternative to RuvC [76]. Additionally, two pathways of RecBCD-mediated recombination have been demonstrated: a “break-join” route that requires the RuvABC Holliday-junction endonuclease system, and a replicative or “break-copy” resolution route that operates independently of RuvABC and RecG [77], [78]. The latter pathway may operate independently of Holliday junctions [78].

To test whether patches, and their strand polarity, result from Holliday-junction resolution, λ by λdv crosses were performed in Δ*ruvC* or Δ*topB* deletion strains. Neither showed significantly different ratios of 5′ to 3′ patches (Figure 7, and Figure 4B, respectively) from the wild-type strain. The data from two experiments each yielded ratios as follows: *rec* ^+^ showed a 0.7±0.5∶1 ratio (mean±range, lower than in previous experiments), Δ*ruvC* showed a 0.9±0.2∶1 ratio. These ratios are slightly lower than those seen in previous experiments, but are not significantly different from each other (*P* = 0.667, by one way ANOVA). In two separate experiments, Δ*topB* showed a ratio of 1.1±0.2∶1 (mean±range), whereas the *rec* ^+^ strain analyzed in parallel showed a ratio of 0.98±0.12∶1. Moreover, surprisingly, patch frequencies show that patches are not dependent on the presence of either RuvC or TopB (Table 3). These data imply that processing of Holliday junctions by RuvC or Topo III is not required for the formation of patches, and suggest that *none* of the three models presented in Figure 1 is likely to bear on patch formation. We shall suggest below that Holliday junctions might not be intermediates in patch formation, and that patches are not derived from the same intermediate(s) as splices. We will propose two different models for the formation of patches, models in which patches form by a fundamentally different mechanism from that leading to splices.

## Discussion

This study employed physical analyses of heteroduplex DNAs using a loop-strand conformation to separate, on polyacrylamide gels, heteroduplex DNAs with an 18 nt loop in either the 5′ or 3′ strand. This method was used to analyze patches formed *in vivo* by RecBCD-mediated DSE-repair recombination of phage λ with the λ-homologous plasmid, λdv, and analyzed patches inserted into the plasmid. Like a previous study of RecBCD-mediated patches analyzed by a different physical method–partially denaturing gel electrophoresis [11]–a prevalence of 5′ and 3′ patches was observed (polarity relative to the DSE where RecBCD loaded, in λ, the λ right end [2]). The results presented here support their conclusion using an independent method, and extend them in the following ways. First, λ Orf and Rap recombination proteins, which were present in previous experiments [11], do not contribute to the relative prevalence of 5′ and 3′ patches (Figure 5), even though they do stimulate recombination efficiency overall (Table 3, and [26], [56], [60]). Second, there is a small but statistically significant shift toward more 5′ patches in *recD* mutants (Figure 6). Third, RuvC Holliday-junction endonuclease, which is required for break-join resolution of RecBC-mediated DSE repair, which reflects Holliday-junction cleavage *in vivo* [77], [78], is not required for patch formation (Figure 7, and Table 3). This implies that patches do not result from endonucleolytic resolution of Holliday junctions, as about half of splices do. Similarly, this implies that none of the previous models in Figure 1 will account for patch formation. The *E*. *coli* topoisomerase III (TopB) also has no effect on heteroduplex-patch polarity or patch formation (Figure 4B, and Table 3).

Other genetic [9], [10], [22], [24], [25] and physical [11], [23], [29], [79], [80] methods for heteroduplex analysis have been used to examine heteroduplex strand polarity of recombinants to elucidate the molecular mechanisms occurring at various steps of recombination. In one physical analysis of RecBCD-mediated splices, the linear DNA substrate was created by *in vivo* restriction digestion of a λ phage to release a linear molecule containing direct terminal repeats [29], [79]. This substrate was then inferred to undergo intramolecular recombination, leading to heteroduplex recombinant formation initiated by pairing and strand exchange of the 3′-ending strand as inferred from the polarity of the splice junctions. A concern in interpreting the results, however, is that the same recombinants would be formed by single-strand annealing (SSA) of the direct repeats after resection of the 5′ ends. RecBCD-mediated recombination is unlikely to proceed *via* an SSA mechanism in most biologically relevant contexts in which it has been studied (*e.g.*; conjugation, phage-mediated transduction, and DSE repair [81]–[83]) and in model recombination studies using λ [84], because in all of these circumstances a single DSE recombines with an unbroken molecule; that is, these are one-ended reactions. The heteroduplex studies mentioned [29], [79] were unusual in employing a substrate with two complementary DNA ends that could produce recombinants by annealing. Thus, it is unclear whether those results apply to most other RecBCD-mediated recombination.

One of the genetic methods [22] used to examine patch polarity found that the recombinant heteroduplex was formed predominantly in the 3′-ending strand, heteroduplex material having “3′ overhangs” in the recombinant molecules. This work examined only splice recombinants, and is compatible with splices formed *via*, *e.g.*, the model shown in Figure 1C [8], though this model does not explain why 5′ *or* 3′ exonucleases are required for splice formation [12], as the split-end model does (Figure 1B).

Another method examined heteroduplex strand polarity using partially denaturing gel electrophoresis of heteroduplexes containing five mispairs [11]. This system, though useful, has been difficult to work with, and required the use of special strains lacking the mismatch repair system to avoid unwanted repair of the heteroduplexes being examined.

The system for heteroduplex analysis described here utilizes several commonly performed methods, with a few modifications, to create, isolate, and detect heteroduplexes formed *in vivo*. This method can be used with many different strain backgrounds to test effects on recombination between λ and λdv.

Several models for recombination depict only 3′ ends invading a homologous duplex, which predicts the formation of 3′ patches only (Figure 1A and C, and [6]–[8], [61], [62]). However, these models failed to account for existing data showing the presence of two different forms of heteroduplex, 5′ patches and 3′ patches [9]–[11]. Two possible general hypotheses can account for the prevalence of both 5′ and 3′ patches. First, either 5′ or 3′ ends might be able to invade a homolog *in vivo* [21], or second, possibly, patch-strand polarity might reflect an entirely different aspect of recombination (suggested below). Evidence that 5′ ends are able to invade a homologous duplex to form D-loops *in vitro* has been well documented [14], [15]. The discovery of *orf* and *rap* in λ, and their potential roles in the recombination mechanism being studied (discussed above), may have led to the assumption that they contributed to the *in vivo* data demonstrating the presence of 5′ and 3′ patches. The results presented here provide evidence that those gene products did not contribute to the presence of 5′ patches seen in previous studies, and imply that 5′ patches, as well as 3′ patches, result from some aspect of RecBCD-mediated recombination.

One possibility for the source of the two different patch polarities involves the exonuclease activity of RecBCD, and the lack of substantive activity after interaction with Chi, or when the strain is RecD^−^ [2], [3], [64], [85], [86]. The helicase activity of RecBC could generate single-stranded DNA ends of both polarities, either of which could invade a homologous duplex and create a heteroduplex patch of one polarity or the other [21] (Figure 1B). One might predict that a strain mutant for RecD might show an increase in 5′ patches relative to 3′ patches because 5′ ends would not be degraded, and a slight yet significant increase in 5′ patches is seen in Figure 6. In addition, strains mutant for RecD have been shown to produce linear multimers of plasmids because the lack of dsDNA nuclease activity allows rolling-circle replication [87]. This might also affect the increase in 5′ patches observed here. This model appears to require endonucleolytic cleavage of Holliday junctions, and though it is still possible for generating splices, it seems unlikely to be relevant to patches, which form independently of RuvC (Figure 7, Table 3).

The lack of dependence on RuvC for patch formation is an important finding. RuvC is the sole demonstrated HJ-cleavage activity present in wild-type *E. coli*. RuvABC are also necessary for break-join RecBCD-mediated splice recombination *in vivo*, which is thought to reflect those recombination events resolved *via* endonucleolytic cleavage [77], [78]. The inability of other proteins to substitute for RuvABC in break-join recombination suggests that Ruv is the only endonucleolytic resolution pathway available to RecBCD-mediated recombination [78]. Thus, the independence of patch formation on RuvC suggests to us that endonucleolytic resolution of Holliday junctions is not required for patch formation. Most recombination models, including all of those considered previously (Figure 1A–C), predict that RuvC should be required for patch formation. *ruvC* mutants are not completely deficient for splice recombination, but exhibit a recombinant frequency that is decreased by half [77], [78], [88]; *ruvC* removes “break-join” splice recombinants specifically and does not alter splice recombinants formed *via* the “break-copy” or BIR (break-induced replication) route [77], [78]. The remaining break-copy recombinant products require the major replicative polymerase, Pol III, of *E. coli*, and were formed only when replication was allowed. The recombinant products in those assays were splices. Recombinant frequencies for the patches examined here for a Δ*ruvC* strain were found not to be different from *rec* ^+^ (Table 3).

Given the surprising lack of dependence of heteroduplex-patch formation on RuvC, and the expected dependence on RecB and RecA, what might be the origin of heteroduplex patches of both polarities? The presence of patches in the absence of RuvC suggests the possibility that a Holliday junction might not be involved in the formation of the heteroduplexes observed here. Additionally, a strictly replicative single-strand gap-filling type of mechanism would result in homoduplex patches, heteroduplex loops would not be observed. Two possible mechanisms that use replication rather than endonuclease to complete patch formation are suggested here.

One possible mechanism for the generation of the heteroduplex patches observed here is synthesis-dependent-strand annealing (SDSA) [89]. SDSA was first described in Drosophila [90], [91], and does not require Holliday-junction-endonucleolytic cleavage for the formation of recombinants. It does, however, require processing of a DNA break, and strand invasion, which could occur *via* RecBCD and RecA, respectively, in *E*. *coli*. In yeast, Allers and Lichten [92] showed that patch formation is differentially timed and patches appear in advance of splices (which require Holliday-junction resolution). This led them to suggest that SDSA produces patched (non-crossover) products primarily, whereas the double-strand break repair mechanism of Szostak *et al*. [93], which includes HJ cleavage, leads to patches and splices (crossovers).

How might both 5′ and 3′ patches be formed *via* SDSA? One possibility is that resection of the 5′ ends at a double-strand break (DSB) leaves 3′-ssDNA ends to invade a homologous duplex (Figure 8). One could imagine that the 3′ end on either side of the DSB could be the invading end, which would result in either a 3′ patch or a 5′ patch (with respect to the right end of the molecule) depending on which 3′ end invaded. When examining a particular locus, such as *ren*, if the break occurred to the left of the marker, a 3′ patch might result, and if the break occurred to the right of the marker, a 5′ patch might result. This model would produce both 5′ and 3′ patches using strand invasions of a single (3′) polarity.

SDSA could also explain previous data reporting the presence of 3′ and 5′ patches [9]–[11]. The approximately 1∶1 ratio observed here is not very different from the approximately 2∶1 ratio reported previously [11], and might be attributable to the difference between the two methods used for heteroduplex analysis. The more substantial 9∶1 ratio reported for a marker in the *P* gene of lambda [9] can also fit the SDSA model as described similarly for the split-end model [21]. The *P* gene of lambda is very close to the origin of replication (*ori*) and perhaps as with phage T4 [94], replication is abandoned more frequently near *P*, causing breaks more often that would need to be repaired. A break would be processed by RecBCD to create 3′ ends which prime synthesis from a homologous DNA molecule. The newly synthesized DNA would create a heteroduplex patch. Perhaps there is a site just to the right of *P* that causes replication fork stalling and breakage such that the template strand of the leading strand, or the nascent strand of the lagging strand, often becomes the invading strand. This would bias patches toward the 5′-ending strand.

Although SDSA has not previously been considered as a recombination mechanism in *E. coli*, it has been studied for years in yeast and Drosophila, and also underlies genome reassembly following high-level double-strand breakage in *Deinococcus radiodurans* [95]. Further work should be able to define any role of SDSA in *E. coli* recombination more clearly.

A significant weakness of the SDSA model is the requirement that DSBs originate in the plasmid. We hypothesize that these might occur at some low frequency due to collapse of stalled replication forks, but we have not shown that DSBs are formed. Nevertheless, we cannot disprove that plasmid-bourne DSBs are formed leading to DSEs that are repaired *via* SDSA.

A more appealing alternative model that does not require DSBs to be formed in the plasmid is shown in Figure 9. This model is based on single-strand assimilation models of Leung *et al.* [96], and Ellis *et al.* [97]; Court *et al.* [98], for yeast and phage λ Red-mediated recombination, respectively. The model in Figure 9 suggests that pieces of ssDNA that are released from the λ chromosome by the nuclease activity of RecBCD are assimilated into one or the other DNA strand of the plasmid during replication. ssDNA fragments that anneal across from the 18 bp insert will create heteroduplex DNA at that site. This model would require RecBCD and RecA, both of which are required for heteroduplex formation observed here. ssDNA oligonucleotides have been used to create mutations by gene targeting in yeast using Rad51, Rad52, and Rad59 annealing activities [99], [100] and in *E. coli* using the phage λ Red beta protein [97], thus demonstrating the plausibility of this model.

One apparent difference between our results with RecABC and those of Ellis *et al.* for Red-mediated recombination, is that whereas we see results consistent with equal incorporation of DNA fragments into either DNA strand, the Red system shows a bias toward incorporations of ss-DNA oligonucleotides into the lagging strand, though this preference is not absolute [97]. This difference might reflect the fact that whereas Red uses a single-strand-annealing protein, beta, the RecABC-dependent recombination studied here uses RecA, which can catalyze both strand invasion and annealing. This might allow single-strand assimilations into the leading strand, where gaps are expected to be less frequent. These assimilations would still require replication and attachment to a new strand during synthesis for completion (per Figure 9). Alternatively, perhaps the RecBCD system also inserts patches preferentially into the lagging strand, but perhaps we see equal numbers of heteroduplex patches in both strands because of the difference between the *E. coli* and λdv replicons. Unlike the bacterial chromosome, λdv does not have a terminus of replication with replication pause sites that block replication forks that go past the “terminus”. Thus, it is possible that replication forks pass across the marker site in both directions, making both strands the lagging strand at some point in the reaction which results not in a bias, but in an equal number of patches in both strands.
